# Towards Millimeter-wavelength: Transmission-Mode Fresnel-Zone Plate Lens Antennas using Plastic Material Porosity Control in Homogeneous Medium

**DOI:** 10.1038/s41598-018-23179-8

**Published:** 2018-03-28

**Authors:** Javad Pourahmadazar, Tayeb A. Denidni

**Affiliations:** National Institute of Scientific Research (INRS), Centre for Energy, Materials and Telecommunication (EMT), Quebec, Montreal H5A 1K6 Canada

## Abstract

We present two transmission-mode dielectric Fresnel-Zone Plate Lens (FZPL) antennas for use within the V-band spectrum. The proposed FZPs are realized via pure plastic material using two different additive manufacturing processes. The proposed FZP lenses are designed with half (*λ*/2) and quarter (*λ*/4) phase correction rings at 60-GHz with 30*λ*_0_ diameter, where *λ*_0_ is the free-space wavelength. The permittivity effect for lens sub-zones is controlled by material porosity in cube-shaped structures. The 3D printed zone plate lenses are built using additive manufacturing plastic materials with a thickness of *λ*_0_ and constant relative permittivities equal to 2.76 and 3.6. Different types of antenna with cos^*n*^-like radiation patterns as lens illuminators are analyzed on the vertical plane of the flat lenses to have a high efficiency over the considered operating band. Simulations and experimental measurements show a reasonably close match, therefore allowing for a reliable predictability.

## Introduction

Control of permittivity is crucial to design of Gradient Refractive Index (GRIN) lenses such as Luneburg lens^1–4^, half-Maxwell Fisheye lens (HMFE)^1^, and Fresnel zone plates^5,6^. Related studies were principally focused on new material combination or material porosity and deforming to control permittivities. Deforming rigid material structures such as air-holes, cube-shaped structures, or material pressing are an apparent material porosity methods to control intrinsic permittivity^1,7,8^. Among GRIN lenses, Fresnel Zone Plates (FZP), due to their planar merit, reduced weight, ease of fabrication, and cost-effectiveness are relatively more attractive structures to achieve high-gain focus in the millimeter and sub-millimeter wave spectrum^5,9–12^. Nevertheless, there are only a few studies on controlling the permittivities of phase correction zones with plastic porosity and experimental realization of the planar focus lens geometries. All investigations revealed hopeful ideas to achieve the following task: the material porosity was utilized to change the intrinsic material permittivity effects in a homogeneous medium, which was then used in controlling the intended permittivity distribution in the lens surface. A phase correction is achieved by sub-zones in homogeneous or inhomogeneous medium to realize Fresnel-zones. Sub-zone phase correctors have been suggested by stepped zones thickness and air-holes in homogeneous zone plates^11–13^, and distinct permittivity configuration is proposed with multi-dielectric concentric rings in inhomogeneous plates^12^. Cube-shaped material porosity urges the need of analysis to implement planar lens schemes. The cube-shaped plastic cells have been introduced and most recently realized by the additive manufacturing processes to control permittivity^14^, which is inaccessible to achieve with conventional fabrication methods.

The subject of this work is the realization of Fresnel zone plate lenses based on material porosity control. We use multi-permittivity distribution with zone plate rules satisfaction to achieve focusing. Due to phase correction sub-zones with respect to the transmission direction, the objective is to find the zone-plates’ geometry using the material porosity method. In order to realize the synthetic permittivity with plastic, the optimum value of cube size is determined through analysis. By employing a proper cube filling ratio, we identify possible geometries in each sub-zones for a focusing lens. Two half (*λ*/2) and quarter (*λ*/4) phase correction FZP lens geometries using Fused Deposition Modeling (FDM)^14^ and Selective Laser Sintering (SLS)^14^ manufacturing process are experimentally realized, which show a close agreement with the simulation results. The contribution of this work is as follows: First, implementation of low permittivity zones with low permittivity cube cells to reduce reflections. Second, homogeneous porous design ZPs. Third, the High efficiency, gain and low side lobe level compared to previous works. Fourth, the low fabrication costs with the fast manufacturing process.

## Fresnel Zone Plate Lens Design

**Figure 1:**
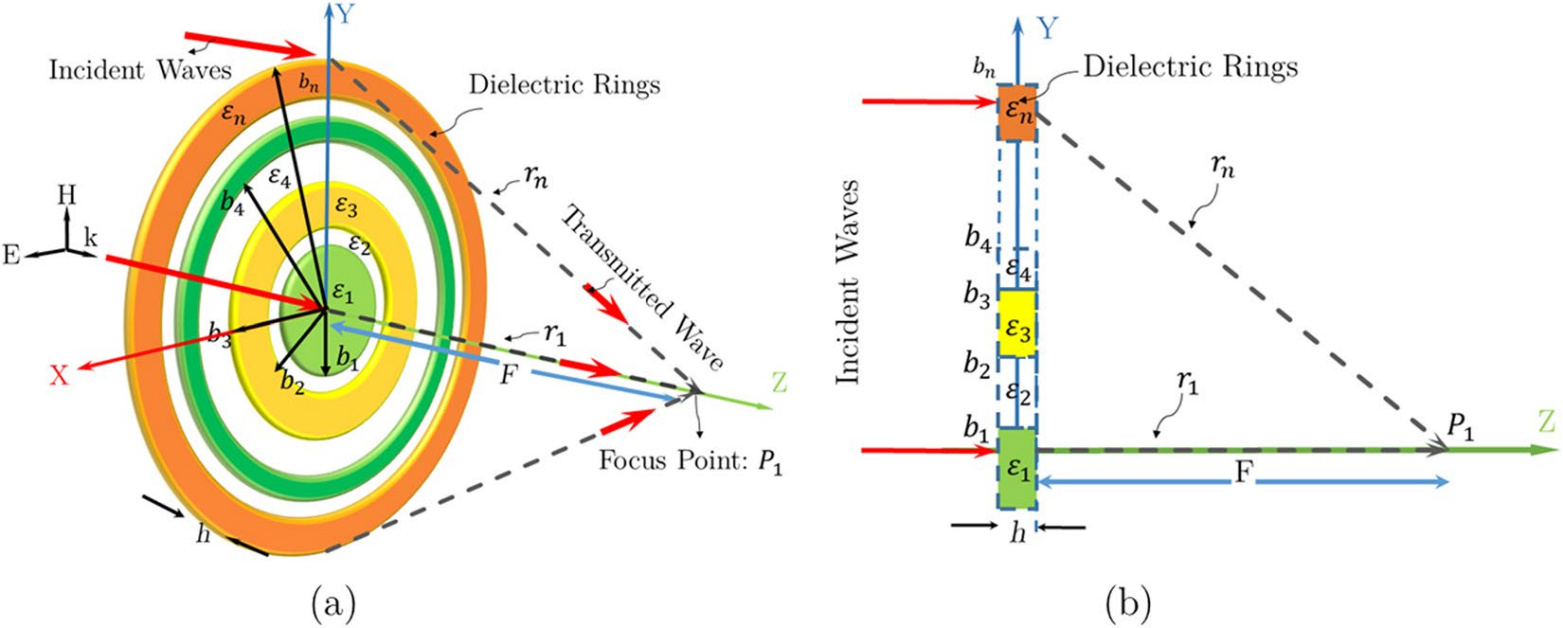
Illustration of the dielectric Fresnel zone plate focusing at *P*_1_ for (**a**) 3D topology and (**b**) half-portion multi-dielectric, phase correcting zone plate in *xy*-plane for perpendicular illumination.

The topology of full dielectric Fresnel zone plate antenna is shown in Fig. 1(a). The FZP lens structure is composed of dielectric rings for phase reversing of incidence waves instead of blocking as Soret type Zone Plates (ZP)^10^. *D* and *F* denote the aperture diameter of the zone plate and distance between the feed and the aperture of the plate, respectively. As shown in Fig. 1(a), Fresnel zone plate is created to transform the incoming wave in transmission mode with a pencil beam in the *z*-axis direction. The Fresnel zone plate (FZP) boundary equation for a conventional planar zone lens with P full-wave circular zones is given by

1 $$\begin{array}{l}{b}_{p}\,=\,{(2pF\lambda +{(p\lambda )}^{2})}^{1/2},\,p\,=\,1,2,\mathrm{..}.,P,\end{array}$$

where *b*_*p*_ denotes the outer radius for the *p*-th sub-zone of the proposed lens, *λ* is the design wavelength at operating frequency, and *F* is the proposed lens focal length. Each full-wave zone in the zone plate (ZP) lens design is divided into an even number of sub-zones, i.e., q = 2, 4, …, 2 *n*. In this ZP lens, the phase at each n-th sub-zone varies from the nearby sub-zone phase by $\pm \frac{2\pi }{q}$ radians^11,12^. The external radius *b*_*n*_ of the *n*-th sub-zone is given by

2 $$\begin{array}{l}{b}_{n}\,=\,\sqrt{\frac{2n\lambda F}{q}+\frac{n{\lambda }^{2}}{q}},\,n\,=\,1,2,\mathrm{..}.,S,\end{array}$$

where *S* is equal to *qP*.

Therefore, with these equations, for the half-wave phase correction *q* = 2, the sub-zones are becoming to the half-wave Fresnel zones, and for *q* = 4, the ZP lens is equal to a quarter-wave phase correction lens^10,12^. In the half-wave zone plate structure, the lens can be a amplitude (binary) or phase-reversal^10^. Therefore, we determine and classify the zone plates lens for *q* = 6, 8, *etc*. with *FZP*_*q*_ subscriptions. The 3D focusing action of the zone plate lens is illustrated in Fig. 1(a). This focusing action in upper half portion of the proposed lens is illustrated by a ray-tracing into 1-st, and *n*-th sub-zones in the first full-wave zone (*p* = 1), where *n* is greater than one. The ray tracing equation for the *r*_*n*_ and *r*_1_ = *F* ray collisions at *p*_1_, as shown in Fig. 1(b), is given by

3 $$\begin{array}{l}(\beta \sqrt{({\varepsilon }_{n})h}+\beta {r}_{n})-(\beta \sqrt{({\varepsilon }_{1})h}+\beta {r}_{1})\,=\,2\pi ,\,n\,=\,2,3,\mathrm{..}.,q,\end{array}$$

where the free space-wave phase constant is *β* = 2*π*/*λ*, *h* is the concentric dielectric ring thickness, and *ε*_*n*_ and *ε*_1_ are the permittivities of *n*-th order, and 1-st order dielectric rings phase shifters, respectively (see Fig. 1). Similarly, with substituting *r*_*n*_ = *r*_1_ + (*n* − 1)(*λ*/*q*) into (3), it becomes

4 $$\begin{array}{l}\sqrt{{\varepsilon }_{n}}=\sqrt{{\varepsilon }_{1}}+(\frac{\lambda }{h})[\frac{1-(n-1)}{q}],\end{array}$$

As shown in Fig. 1(b), for a perpendicular illumination the plane wave goes into the first zone without any reflection, if the proposed lens meets the standing wave condition as Eq. 4. Since, the dielectric ring thickness *h* is measured by half-wavelength of the n-th standing dielectric, where n is the integer number, the ${\lambda }_{{\varepsilon }_{1}}$ for the first sub-zone will be equal to *λ*.

5 $$\begin{array}{l}h\,=\,\frac{n\lambda }{2\sqrt{{\varepsilon }_{1}}}\,=\,\frac{n{\lambda }_{{\varepsilon }_{1}}}{2}.\end{array}$$

Finally, by replacing equations (4) into, (5) the final dielectric zone plate mathematical equation is obtained as follows

6 $$\begin{array}{l}{\varepsilon }_{n}\,=\,{\varepsilon }_{1}.{[1+\frac{2}{n}(1-\frac{n-1}{q})]}^{2}.\end{array}$$

The thinnest designed lens thickness corresponds to *n* = 1, which is given by *n* and *q*. Accordingly, with increasing *n*, the proposed ZP lens thickness *h* will be proportional to *n* (i.e., *h* ∝ *n*) and permittivity ratio *ε*_*n*_/*ε*_1_ will decrease. In the proposed design, we expand on previous multi-dielectric zone plates to provide the new result which is achievable with virtual permittivity for phase corrector design at the millimeter-wave spectrum. The design graphs for the zone plates are analyzed based on 3D printing^14^ material porosity with the electromagnetic model and measurement. In order to show the designed lens focusing ability and geometrical properties with virtual permittivities. The additive manufacturing process is chosen to produce these models. A Cube-shaped porosity model is described to provide necessary permittivity feature. For this reason, an optimum size of cubes is achieved through a full-wave analysis in combination with verifying results.

## Permittivity Control with Material Porosity Method for ZP Zones

**Figure 2:**
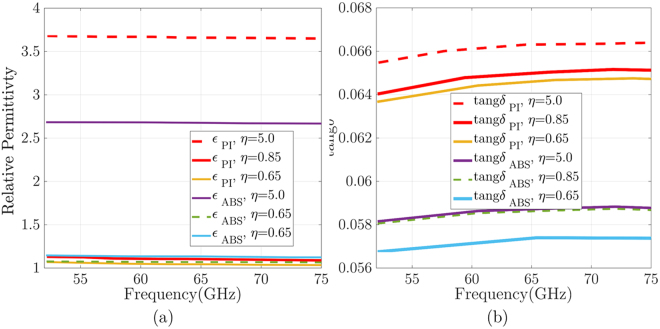
Illustration of V-band characterization for ABS, and Polyimide 3D printed samples: (**a**) relative permittivity (*ε*_*r*_) and (**b**) Loss tangent; for 3D printed materials extracted from the measured S_11_ and S_21_-parameters with Kramers-Kronig relation.

**Figure 3:**
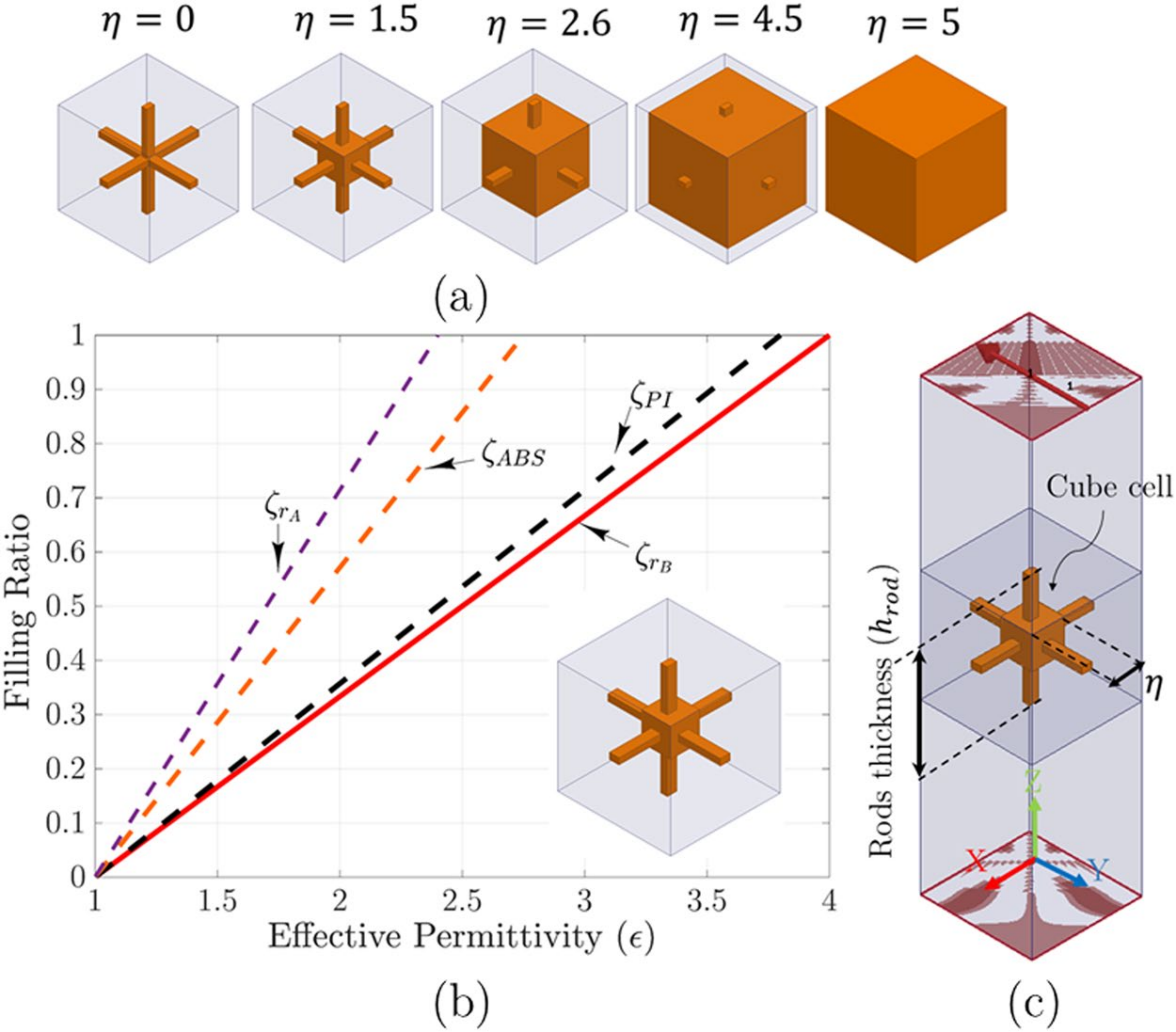
(**a**) Illustration of the filling ratio *ζ* vs. effective permittivity for ABS, Polyimide, and two materials with close permittivities ${\varepsilon }_{{r}_{A}}=2.4$, and ${\varepsilon }_{{r}_{B}}=4$ for the filling ratio $\zeta$ extracted from (1). (**b**) HFSS simulation setup for effective permittivity analysis, where ${h}_{rod}=5\,mm$ is the thickness of rods, and *η* is the size of cube 0 to 5 mm.

The cube-shaped material porosity techniques were utilized with additive manufacturing process to change the intrinsic material permittivity effects in a homogeneous medium, which is impossible with previous manufacturing processes. We expanded this porosity model to satisfy the expected relative permittivity to produce phase reversing of planar zones. The analyzed cube cells are used to realize entire zone plate volume with discrete and separate cells. Overall cube-shaped cells are constructed with two distinct polymer-based plastic, which is chosen from EOS additive manufacturing systems and materials^14^. Since the accurate permittivity of the proposed dielectric materials is crucial to simulate, design and fabricate millimeter-wave GRIN medium ranges efficiently. Therefore, both materials (ABS-M30, and Polyimide) waveguide fill samples with dimensions of 3.7 × 1.8 × 5 mm^3^ are built to fill WR-15 waveguide spacer. Then an Agilent E8361A PNA Network Analyzer is used to enable V-band measurements of the ABS-M30, and Polyimide dielectric samples. The Kramers-Kronig (KK) relation is used to extract printed material characteristics (the relative permittivity and loss tangent) from the measured S_11_ and S_21_-parameters. Figure 2 shows the extracted measurements of relative permittivity (*ε*_*r*_) and loss tangent (tang *δ*) for the printed samples over the V-band. As shown in Fig. 2, the relative permittivity *ε*_*r*_ measurements appear stable and fairly linear for frequencies up to above 70 GHz with a small downward slope as frequency increases. As expected, PA2200 nylon (Polyimide) SLS material exhibits a higher permittivity. The variation of the *ε*_*r*_ measurements for the Polyimide material are likely the result of dimensional variations for the waveguide fill samples due to the developing tuning of the processing conditions for this nylon. Currently, relative permittivity measurement variations of less than ±3.2% and ±6% are achieved for the ABS-M30 and Polyimide samples, respectively. Loss tangent (tan *δ*) measurements yield maximums of 0.059 and 0.068 for the ABS-M30 and Polyimide samples, respectively, demonstrating their suitability for millimeter-wave applications. The full size of these cell for both dielectric materials is 5 × 5 × 5 *mm*^3^, as shown in Fig. 3(a), which *η* is the cube vertex size. These cells are connected with rectangular rods as a mechanical supporter to realize entire plates, as shown in Fig. 3(a). The dimension of rod connector is fixed at 0.65 mm diameter to have a little impact on the zone plates focusing ability. By tuning each cube vertex size *η*, the expected dielectric constant is produced. As we expected in this material porosity model, by reducing the cube volume in control of the filling ratio (*ζ*), a lower effective permittivity compared to the full cube size is achievable. To achieve *q* zone geometry, the predetermined filling ratio (or cube sizes) are used to realize each zone based on the permittivity distribution given by (6).

Since the employed relative permittivity for ABS-M30^14^ and Polyimide^14^ are 2.76 and 3.6, which is achieved with measurement, the desired permittivity realization within one up to those permittivities through filling ratio control is easy. The full cube size for this analysis is 5 mm, which is equal to *λ*_0_ at 60 GHz.

**Figure 4:**
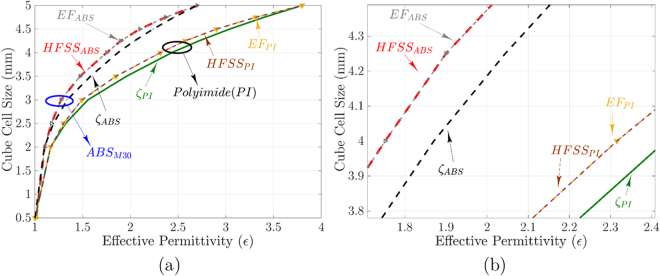
(**a**) Illustration of the Cube size vs. effective permittivity analysis for ABS-M30 and Polyimide plastics with filling ratio *ζ*, exponential fitting (*EF*), and Ansoft HFSS simulations. The *EF*_*ABS*_, and *EF*_*PI*_ curves are the extracted data from exponential fitting function. The ABS polymer cube vertex size *η* for intended permittivity is obtained using exponential fitting (*EF*) equation, where $\eta \,=\,5.545-58092$ × ${e}^{-{\varepsilon }_{r}/0.07564}-9.5423\times {e}^{-{\varepsilon }_{r}/0.95527}$, *ε*_*r*_ is the intended permittivity, and *η* is the cube vertex size for ABS plastic cubes, (**b**) Zoomed for 1 ≤ ε ≤ 2.4.

As shown in 3D volume reduction scheme in Fig. 3(a), each cube is formed with air voids. In this scheme, by assuming effective permittivity and filling ratio as a set of data points such as (*x*_0_ = *ε*_*Air*_,*y*_0_ = *ζ* = 0), (*x*_1_ = *ε*_*m*_,*y*_1_ = *ζ* = 1), a new data points using linear interpolation is obtainable. Therefore, a cube-shaped cell effective permittivity with volume reduction is approximated with linear interpolation given by

7 $$\begin{array}{l}{\varepsilon }_{r}\,=\,{\varepsilon }_{m}.{\zeta }_{m}+{\varepsilon }_{Air}.(1-{\zeta }_{m}),\end{array}$$

where *ε*_*m*_ is the material permittivity, which *m* is dedicated to ABS-M30 and Polyimide plastic materials, whereas *ε*_*Air*_ stands for the air permittivity. Figure 3(b) shows the filling ratio *ζ* vs. effective permittivity results. As shown in Fig. 3(b), the filling ratio versus effective permittivity analysis for *ε*_*rA*_ = 2.4 and ABS-M30 even with close permittivities are so far. Moreover, raw material intrinsic permittivity is essential for a final cube size. For this reason, the distinct analysis should be considered for each particular substance individually. The extracted results for cube size vs. filling ratio shows that the cube size variation vs. effective permittivity is not linear. In order to obtain an acceptable design tolerance, we cannot confine just for this approximation to create the zone plate lenses. In a parallel study, an Ansys HFSS simulation setup is used to calculate the optimum value of cube size to reach this goal. Each ABS-M30 and polyimide plastic cube-shaped cells with rods are analyzed in the waveguide, with PMC and PEC boundaries for setting up the periodic environment. For this setup, the wave ports are located on the top and bottom of the cube cell, as shown in Fig. 3(c). The effective permittivity of each cube-shaped cells is obtained from scattering parameters using the standard retrieval method. As shown in Fig. 4(a), the extracted results of cube size vs. effective permittivity for filling ratio *ζ* and HFSS results are similar up to 2.2 mm. To solve these uncorrelated explanations, the exponential fitting method is applied to extracted cube sizes, as shown in Fig. 4(a). By this fitting, the *EF*_*m*_ results matched well with *HFSS*_*m*_ as HFSS setup outs, where *m* is the material types. To review, the realized lens cube sizes would be the HFSS and EF outs, as shown in Fig. 4(b).

**Figure 5:**
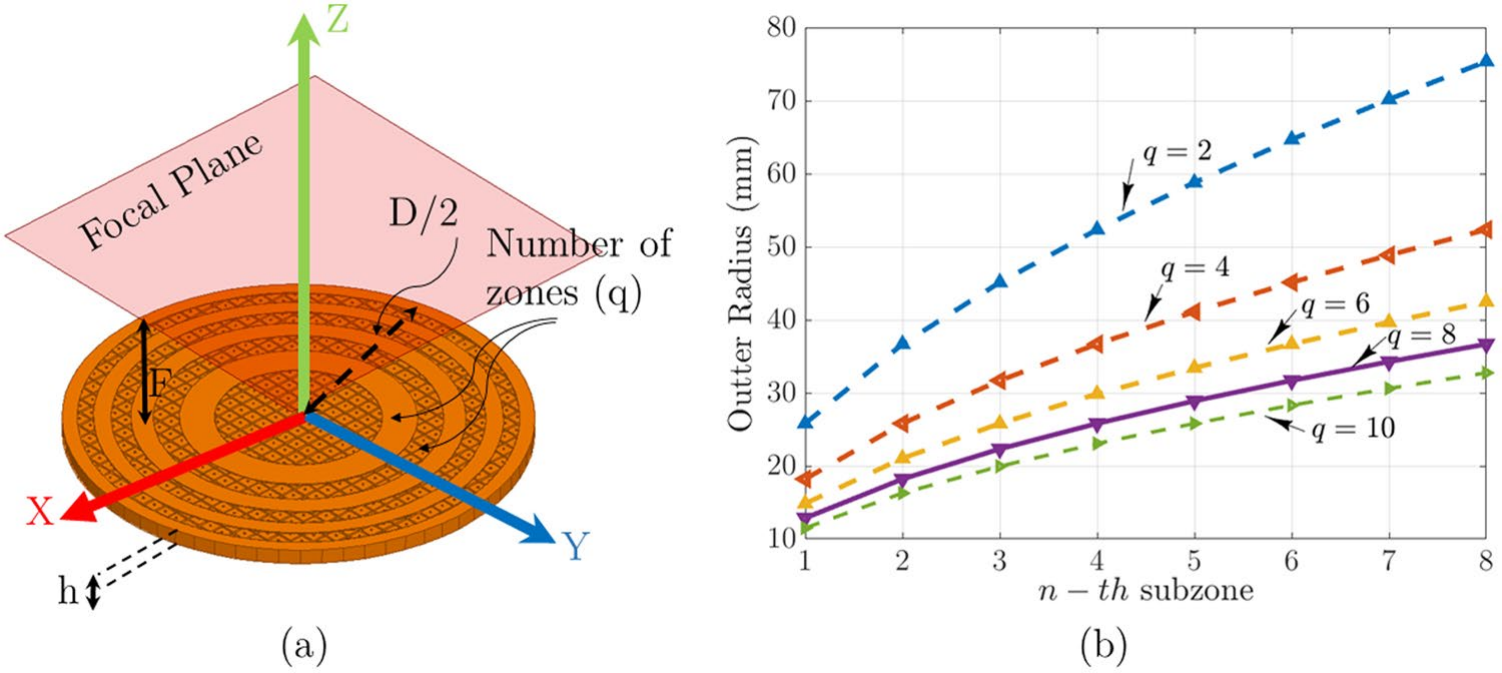
(**a**) The primary geometrical parameters that determine our lens structure are lens diameter *D*, focal length *F*, and the number of zones *q* (for this figure *q* is 2); (**b**) External radius of the Fresnel zone plate versus sub-zone for 2 ≤ *q* ≤ 10 with *λ* = 5 *mm* given by (2).

After parametric studies to extract optimum size using cube size versus effective permittivity analysis, the focusing ability of phase-correcting zones with cube-shaped cells was performed by commercial software CST Microwave Studio. The values of cube size were chosen based on typical ranges used for lens sub-zones given by (6) for millimeter-wave zone plate antenna implementation. The four primary geometrical parameters that determine our lens structure are cube size *η*, lens diameter *D*, focal length *F*, and the number of zones *q*, as shown in Fig. 5(a). For zone plate design, *D*, *F*, and *q* are interrelated through (1). Therefore, we can choose just two sets of these parameters independently. Some interesting trends that are apparent based on (1) in our analysis which can highlight as guidelines for any design as follows. By increasing the zone plate diameter *D* and holding the focal point *F* constant, the lens focusing gain will be increased. This difference indicates that to have a more focus on incident energy with lens rules (1) satisfaction the higher number of zones should be enabled. However, this increase of the focusing gain is limited, which is prognosticated with geometrical optics. In order to increase *D*, the outer zone widths relative to the operating frequency wavelength will be thinner, as shown in Fig. 5(b), which makes the designed plates inaccurate. However, if we held the lens focal point and diameter variable, and number of zones constant with Equation (1), the lens focusing gain will improve like before, but this enhancement will not raise unbound because of the multiple reflections by high permittivity zones. Therefore, to obtain a good focusing gain in our plates, all these trends are considered during the design.

In this study, the zone numbers *q* ranges from 1 to 10 but the lens focal length and diameter are fixed at 26*λ*_0_, and 35*λ*_0_, respectively. As mentioned before, with increasing of zone numbers, the lens surface is subdivided into thinner width zones, as shown in Fig. 5(b) analysis given by (2). For this reason, the realization of high *q* lenses with 5 mm cells will be a critical design point with the implementation of the porosity cells. Therefore, only half and quarter wave phase correction is selected to evaluate the performance of zone plates with this porosity technique. After choosing the zone number, the entire lens surface is subdivided into 5 mm cells, which is divided plates surface to 900 unit cells. Then a predetermined cube size based on the parametric permittivity analysis, as shown in Figs 3(b) and 4(a), is chosen to fill these cells to realize the entire lenses. As mentioned before, the multiple reflections of the plate because of the high permittivity zones is conventional in the zone plate structures. To reduce these reflections in our plates, a thicker lens (*t* = *λ*) using smaller effective permittivity zones is designed to produce intended phase shift with lower attenuation. There are three reasons for the choice of this thickness. First, this increased thickness (*t* = *λ*) allows us to reduce the multiple reflections of high permittivity zones. Second, the implementation of low permittivity zones with low permittivity cube cells will be possible. Third, the designed lenses will achieve the required mechanical resistance due to the spatial state of construction. Therefore, the design of half and quarter-wave Fresnel zone plates using cube-shaped material porosity at 60 GHz will be possible. The proposed lens radiation system was simulated using the finite difference time domain method software CST.

Lens design criteria for the focusing devotions can be classified into two modes of illumination: transmission and reflection modes. The essential devices of each focusing setup are assigned to two central parts that can achieve them in all pieces of literature^1–19^ with specific names: a lens device (focusing provider) and an illuminator/feeder/wave-launcher. Generally, all classes of lens devices for wave-focusing setup are independent of electromagnetic spectrums, and they have had their treatment scenarios and produce predetermined sub-zone permittivity techniques. However, the treatment of the proper illuminator and low-error subzone permittivity implementation is the apparent goal of all focusing mechanisms, which can be considered to guarantee the desired radiation effect in a predetermined setup with a defined focal point (F), lens diameter (D), and antenna gain parameters.

As shown in Fig. 1(a), a Fresnel zone plate is a planar symmetric gradient index structure with a z-axis in the center. Therefore, by providing axially symmetric illumination^9^ for feeding lens apertures, the zone plate’s radiation patterns should be symmetrical with high gains and high efficiency at the focusing point (P1). Therefore, we can verify that any asymmetrical zone plate’s radiation is due to permittivity estimation errors in the homogeneous design principles. To achieve this goal, an axially symmetric feed^6^ or uniform feed^15,19^ based on the^15,19^ feed analysis is considered.

In millimeter-wave band over 30 GHz, the fabrication of illuminators that uniformly^15,19^ or axially symmetric form illuminates^6^ the lens surface to obtain an intended efficiency is difficult^19^. For this reason, amplitude tapering must be considered for illumination of lens feed to obtain the desired efficiency for proposed lenses based on^15,19^ feed analysis. To accomplish this goal, two crucial factors are considered based on Kildal feed analysis, and approximations in^15^: the taper efficiency (${\eta }_{taper}$) and the spill-over efficiency (${\eta }_{SP}$)^15,19^. In the classic design of Fresnel zone plates, horn antennas are employed as an illuminator to feed the zone plates, the radiation pattern of the lens feeder serves a cos^n^-like function^6,15,19^. Since the fabrication of this kind of feed at millimeter-wave band is complicated^19^. Therefore, a new type of illuminator or cos^n^-like radiation pattern horn with low SLL must be considered for illumination^6,15,19^. To reach this purpose, theoretical aperture efficiency versus cos^n^-like radiation pattern amplitude weighting is analyzed to obtain expected efficiency with *n* variations^15,19^. The taper efficiency $({\eta }_{taper})\,$equation for the loss of non-uniform illumination of the aperture amplitude and the spill-over efficiency (${\eta }_{SP}$) are given by (Eq. 8) and (Eq. 9), individually^15,16,19^:

8 $${\eta }_{taper}=\frac{1}{\pi .{\tan }^{2}(\frac{{\theta }_{0}}{2})}.\frac{{[{\int }_{0}^{2\pi }{\int }_{0}^{{\theta }_{0}}{|G(\theta ,\phi )|}^{0.5}.\tan (\frac{\theta }{2})d\theta d\phi ]}^{2}}{{\int }_{0}^{2\pi }{\int }_{0}^{{\theta }_{0}}|G(\theta ,\phi )|.\,\sin (\theta )d\theta d\phi }\,,$$

9 $${\eta }_{SP}=\frac{{\int }_{0}^{2\pi }{\int }_{0}^{{\theta }_{0}}G(\theta ,\phi ).\,\sin (\theta )d\theta d\phi }{{\int }_{0}^{2\pi }{\int }_{0}^{\pi }G(\theta ,\phi ).\,\sin (\theta )d\theta d\phi },$$

where G(*θ, ϕ*) is the radiation pattern, *θ* is dedicated to polar angle, and *ϕ* is the azimuthal angle^15,16^.

10 $$\begin{array}{c}G(\theta ,n)=\{(2n+1).co{s}^{n}(\theta ),\,for\,0 < \theta  < \pi /2;\\ \,\,\,\,0,\,for\,\,\theta  > \frac{\pi }{2};\end{array}$$

**Figure 6:**
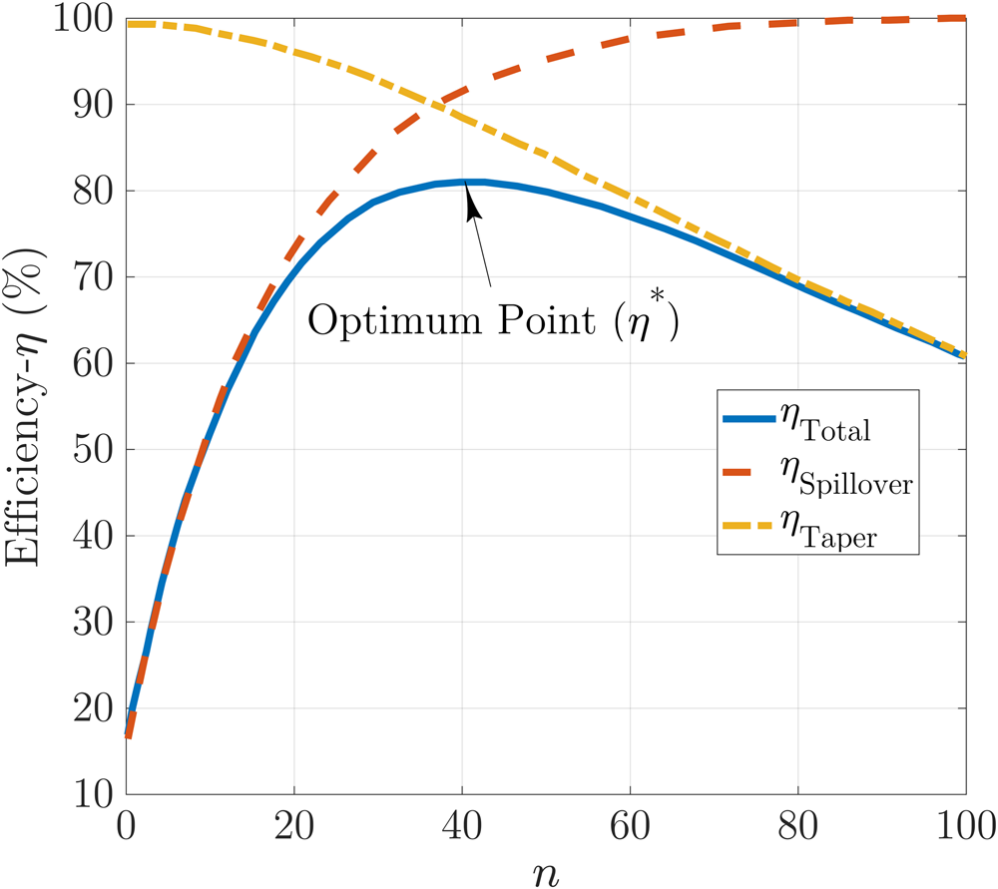
Graphical illustration of *η*_*taper*_ (Eq. 8), *η*_*SP*_ (Eq. 9), and *η*_*total*_ efficiency^19^ over the amplitude weighting generated by cos^n^-like illumination^6,19^.

Since the axially symmetric radiation pattern for illuminator is given by (Eq. 10)^6^, accordingly, with increasing n, the proposed feed will generate high spill-over efficiency (${\eta }_{SP}$), and the taper efficiency (${\eta }_{taper}$) will decrease^19^. Considering to the lens diameter and the lens focal length (F), a higher *n* for cos^n^-like radiation pattern to obtain a product of two efficiencies as total efficiency (${\eta }_{T}={\eta }_{taper}.{\eta }_{SP}$) is desirable^15,19^. Illustration of this efficiency versus *n* value, as shown in Fig. 6, shows that the optimum point (${\eta }^{\ast }$) to obtain maximum total efficiency ${\eta }_{T}$ = 0.81 is equal to *n* = 40^15,19^. As shown in Fig. 6, to obtain the total efficiency between 0.31 < ${\eta }_{T}$ < 0.71, *n* must be chosen between 10 < n < 20 ranges. Considering to provided information for total efficiency higher than ${\eta }_{T}$ = 0.71, *n* must be chosen between 22 and 78. For this purpose, two types of feed as illuminators to study of the designed FZPs efficiency are considered: (a) a cos^10^-like radiation pattern feed, and (b) a cos^45^-like radiation pattern feed. To achieve the first feed with cos^10^-like radiation pattern a microstrip dipole antenna is designed, and to obtain second feed with cos^45^-like radiation pattern a commercial horn antenna are considered to feed lens.

Concerning structural comparison with^19^, the provided lenses do not have any structural similarities or physical relationships and are just determined by the classification of their Fresnel zone plate arrangements with different applications toward two distinct bodies: planar and corrugated forms. In the case of feed types and illuminator designs, all Fresnel lenses analyses with horn antenna illuminations are similar, which is illustrated in^6^ for high-frequency treatments in detail. The presented study in^6^ describes the high-efficiency lens treatments while focusing on the type of illuminations such as axially symmetric feeds (Eq. 9) and lens classes. Although based on^6^ studies, the results of both hard and soft material lenses are foreseeable, but we and^19^ designers tried to solve manufacturing zone plate difficulties with different soft and hard plastic materials. In^19^ Fresnel zone prototypes, the designers have decided to use high-efficiency luminosity in the feeding section at the expense of manufacturing problems and maintenance costs in lens platform to achieve high efficiency. However, the general purpose of our structure is concentrated to producing a cubic cell with the ability of intended permittivity control in a homogeneous environment, which is entirely dissimilar and innovative for Fresnel lens treatments.

Based on two types of radiation feed applied to both lens surfaces, as described in the next sections, lenses-out radiation has an entirely symmetrical form, a high gain, and high efficiency. Expected results compared to previously reported devices with similar type feeds, as shown in Table 1, indicate the accuracy of the estimated permittivity method for phase corrector zones designed with hard plastic cube-shaped cells. Regarding the mentioned results with two distinct materials, the proposed design scheme has already answered the earlier problems with low efficiencies in hard plastic slabs (see Table I). Also, it has responded to the issues of manufacturing and keeping in services for the similar Fresnel-type lenses in^19^ with soft foam materials.

**Figure 7:**
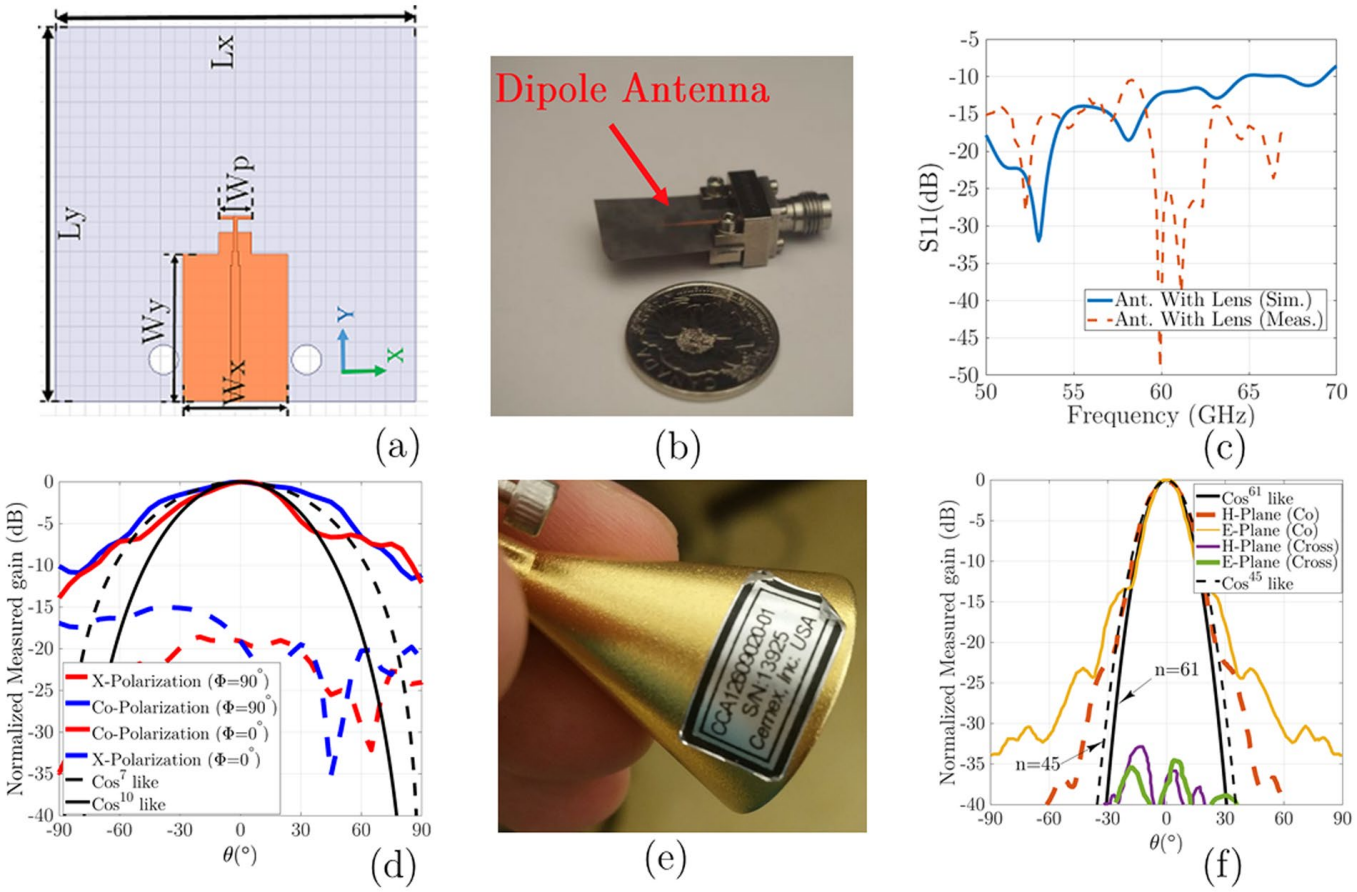
(**a**) Geometry of the proposed dipole antenna: *L* = 12, *R* = 6, *L*_*x*_ = 24, *L*_*y*_ = 24, *W*_*x*_ = 7, *W*_*y*_ = 9.8, and *W*_*p*_ = 2.2 (All in mm), (**b**) Fabricated dipole antenna; (**c**) The measured and simulated return loss for dipole antenna; (**d**) Measured radiation patterns for the dipole antenna and comparison with the associated cos ^*n*^ like pattern at 60 GHz; (**e**) A Commercial conical horn fed by WR-15 waveguide with UG-385/U flange; (**f**) Comparison of the conical horn antenna measured radiation pattern along *ϕ* = 90° (*yz*-plane) and *ϕ* = 0° (*xz*-plane) planes with the associated cos^*n*^ like pattern.

In the first prototype, a microstrip dipole antenna because of the easy and cheap manufacturing process is used to illuminate phase plates surface. For this purpose, the dipole antenna is designed to illuminates plates on *xy*-plane with radiation at *z*-direction and antenna parameters were optimized to have cos^10^-like radiation patterns with optimized antenna parameter. The proposed dipole antenna geometrical dimensions are plotted in Fig. 7(a,b). The simulated and measured results show a good impedance bandwidth over the operating band, as shown in Fig. 7(c). Measured radiation patterns for the dipole antenna in *ϕ* = 0° and 90° planes at 60 GHz are plotted in Fig. 7(d). As shown in Fig. 7(d), the measured E- and H- planes radiation patterns are matched with associated cos^*n*^- like radiation pattern with *n* = 7 and 10 at 60 GHz. These results certify that the determined *η*_*taper*_ theoretically for antenna is valid.

In the second prototype, a Cemex Millimeter, Inc. commercial V-band horn antenna is used to illuminate phase plates surface on *xy*-plane, as shown in Fig. 7(e). The proposed antenna is a conical horn that operates from 58 to 68 GHz with 41 mm length and aperture dimensions of 40 mm. The horn is fed by a WR-15 waveguide with UG-385/U flange. This horn provides a 20 dB nominal gain and a typical half-power beamwidth of 16° in the E (*xz*)-plane and 20° in the H(*yz*)-plane. The horn also offers a typical side lobe level of −20 dB on the E-plane and −28 dB on the H-plane, respectively, with x-polarization level lower than −23 dB. The measured voltage standing wave ratio (VSWR) is 1.15:1, over the frequency range. Figure 7(e) show the proposed conical horn antenna. Measured radiation patterns of the conical horn antenna in *ϕ* = 0° (*xz*-plane) and 90° (*yz*-plane) planes at 60 GHz are plotted in Fig. 7(f). As shown in Fig. 7(f), the measured E- (*xz*-plane) and H-(*yz*-plane) planes radiation patterns are matched with associated cos^*n*^- like radiation pattern with *n* = 61 and 45 at 60 GHz. These results certified that the determined *η*_*taper*_ theoretically for this horn antenna is valid with slightly high *η*_*SP*_ because of the side lobe level.

**Figure 8:**
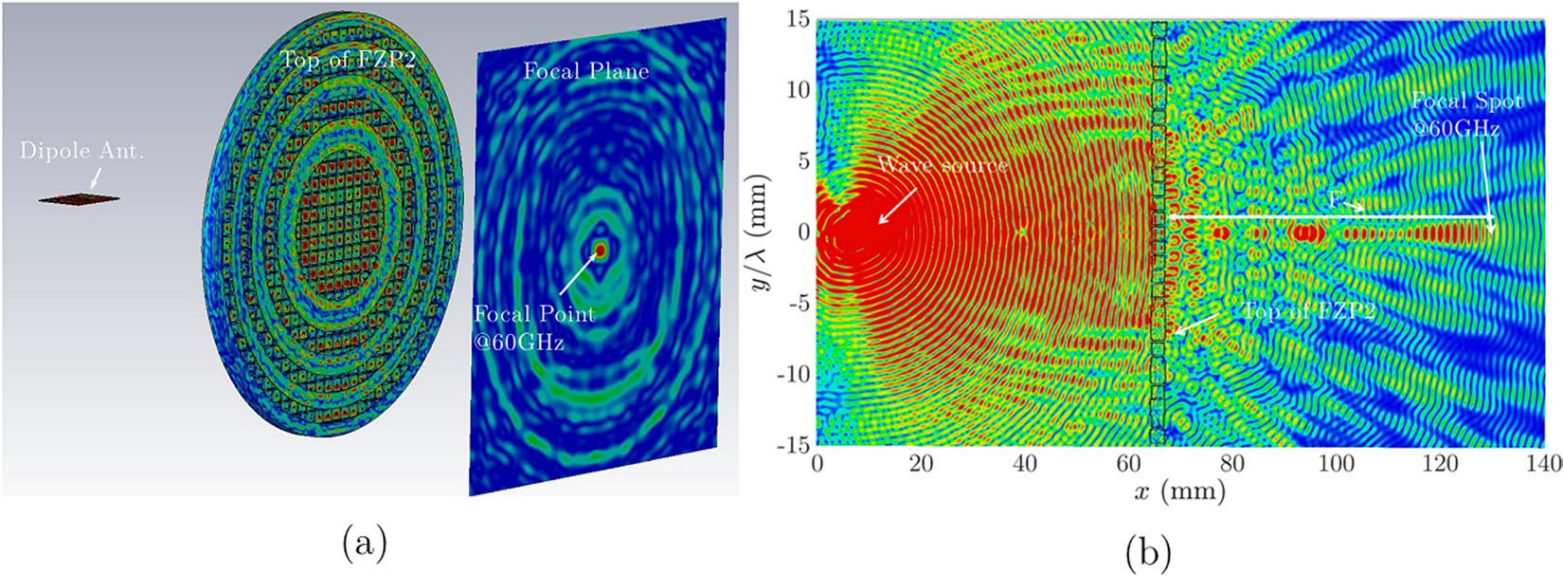
(**a**) Antenna setup for FZP antenna with E-field distribution on Lens surface and Focal plane; Longitudinal radiation output beam at the main focal point with dipole illuminator on *yz* – Plane.

**Figure 9:**
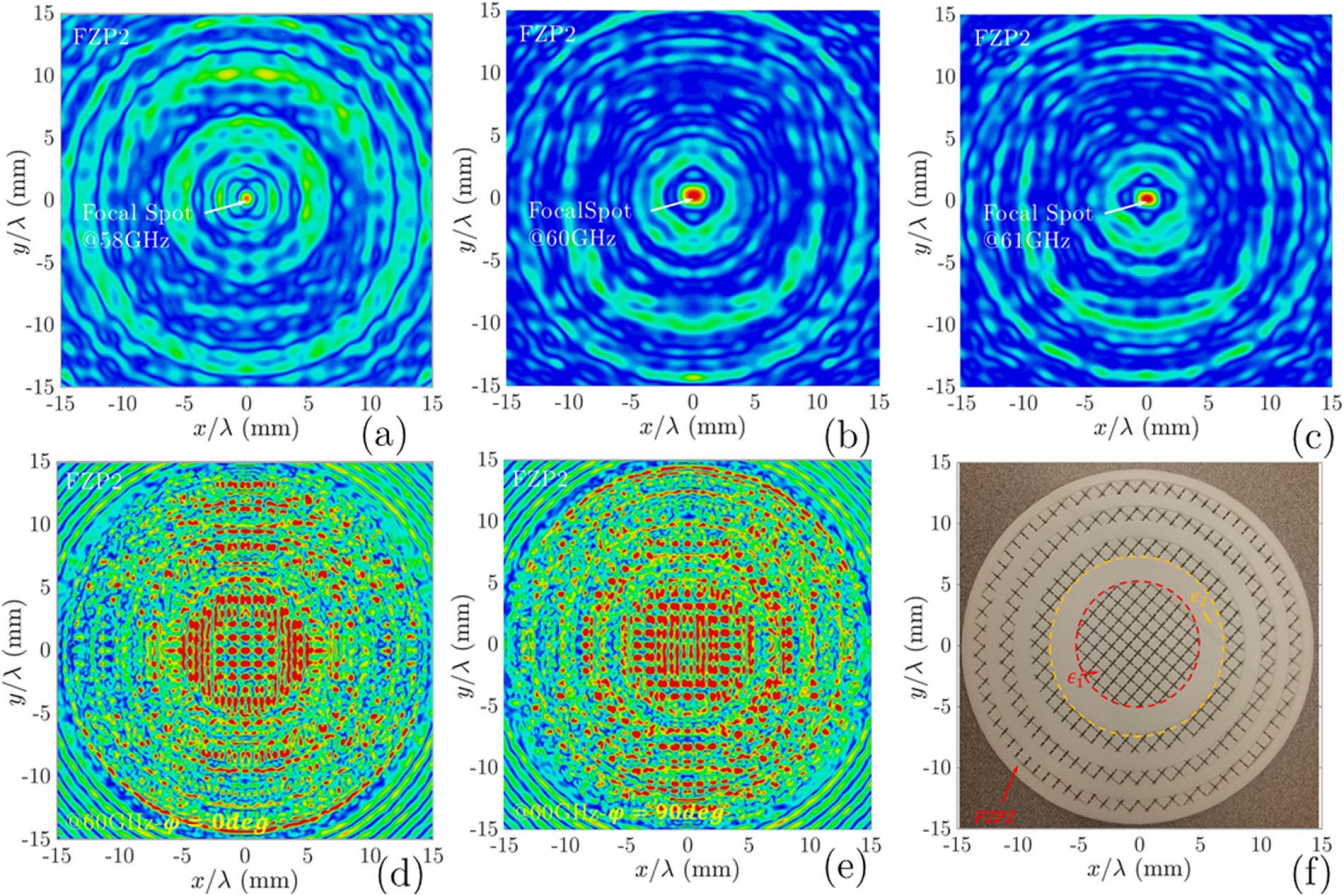
Transversal radiation output beam at the main focal point with dipole illuminator at: (**a**) 58 GHz; (**b**) 60 GHz (**c**) 61 GHz; Transversal |*E*|-field outputs for *FZP*_2_ surface with dipole illuminator: (**d**) *ϕ* = 0°, (**e**) *ϕ* = 90°; For the corresponding plots all data are normalized with maximum values. (**f**) The realized dielectric *FZP*_2_ lens with ABS-M30 using FDM method.

Sequential cross sections (*xz*-plane) for electric field distribution were analyzed in CST set up for a focal plane as shown in Fig. 8(a,b). Using CST software, 3D view of electric field distribution on the focal plane and lens surface is prepared to have the transversal and longitudinal |*E*|-field distribution as shown in Fig. 8(b). Observing longitudinal representations of the electric intensity, the *FZP*_2_ lens focal spot is narrow. Regarding this result in Fig. 8(b), the focal distance is located at 132 mm for both feeder. The *FZP*_2_ lens output radiation beams on focal plane are presented in Fig. 9(a)–(c) at 57, 60, and 61 GHz, respectively. As shown in these figures, the successful conversion of waves in a focal spot at 57 GHz, 60 GHz, and 61 GHz is observed. In Fig. 9(d),(e), the transversal |*E*|-field distribution on *FZP*_2_ top surface for *ϕ* = 0° and *ϕ* = 90° are depicted. In this case, it shows the influence of the number of zones with phase change on the top surface of *FZP*_2_. The photograph of the dielectric *FZP*_2_ model is shown in Fig. 9(f), which is obtained by ABS-M30 polymer plastic with *ε*_*r*_ = 2.76, and *tanδ* = 0.059.

**Figure 10:**
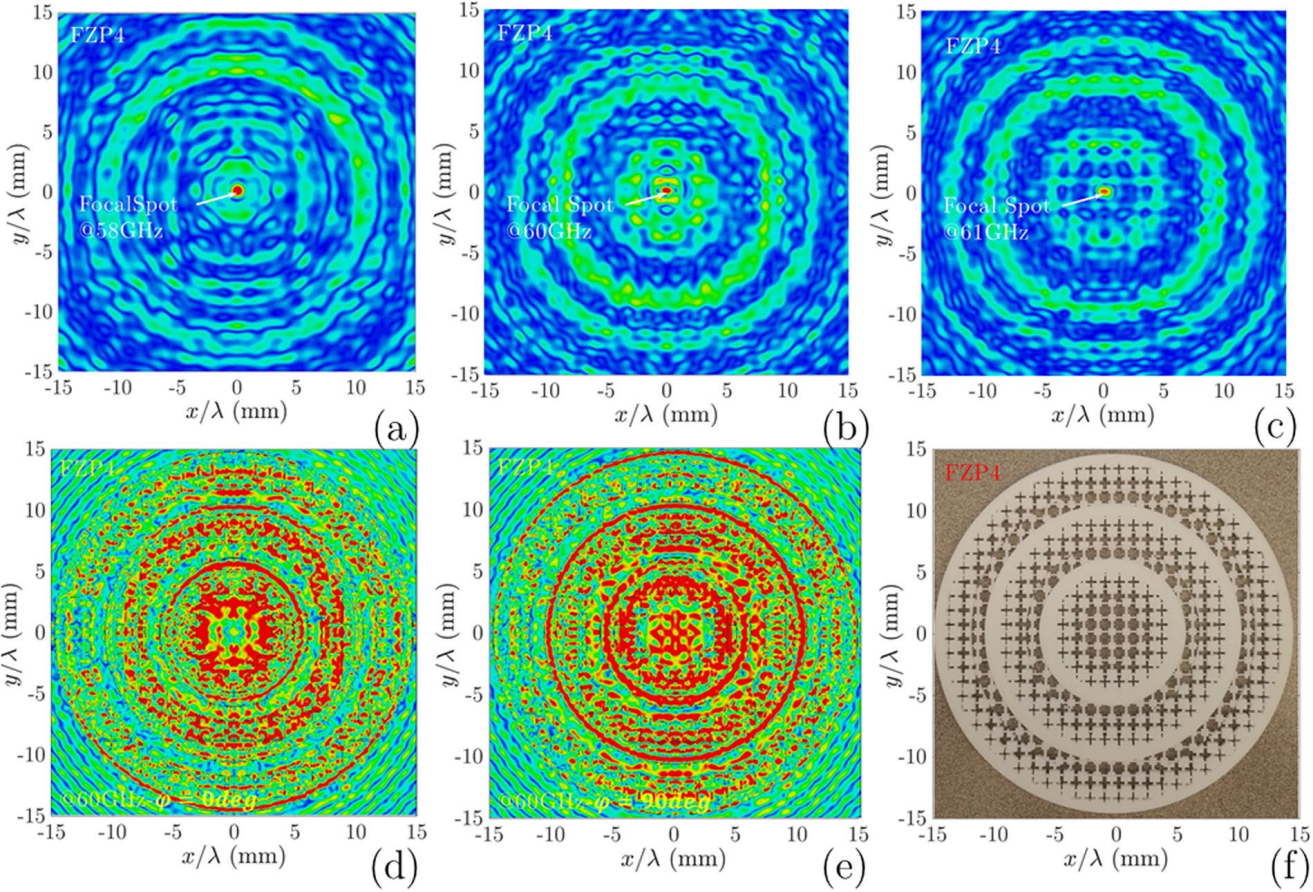
Transversal radiation output beam for the main focal point with dipole illuminator at: (**a**) 58 GHz; (**b**) 60 GHz (**c**) 61 GHz; Transversal |*E*|-field outputs for *FZP*_4_ surface with dipole illuminator: (**d**)*ϕ* = 0°, (**e**) *ϕ* = 90°; For the corresponding plots all data are normalized with maximum values. (**f**) The manufactured whole dielectric *FZP*_4_ lens with polyimide, using SLS method within twelve sub-zones, which is designed by material porosity reduction technique.

In Fig. 10(a–e), the simulated transversal profile for *FZP*_4_ at the focal points are presented. The similar setup is used for the 3D view of the electric field distribution on the focal plane and lens surface. For *FZP*_2_, the radiation beam has a 2.4° width, whereas for *FZP*_4_ is 2°. This result shows the focusing of the wave is stronger for *FZP*_4_ since the plate is composed more diffractive rings. In Fig. 10(d),(e), the transversal |*E*|-field distribution on *FZP*_4_ top surface shows the influence of the number of zones with phase change for *ϕ* = 0° and *ϕ* = 90°, respectively. This |*E*|-field distribution for phase changes shows the effect of cube-shaped permittivity distribution of sub-zones. For the corresponding plots, all data are normalized with the maximum values. The analysis of the radiation beams of the *FZP*_2,4_ phase plates follows a similar approach.

### Half-wave (*FZP*_2_) and Quarter-wave zone plate (*FZP*_4_) Design Method

Two dielectric *FZP* lens antenna were fabricated using an additive manufacturing process including two different type plastic materials. To attain the best performance of the lens design, simulation analysis of dielectric FZP lens results by CST software was accomplished. Our review shows that the plastic lens structures with material porosity simplify the selection of the relative permittivity for lens fabrication in a homogeneous composition, yet with some design limitations. In some particular models, cube-shaped material porosity is examined to implement phase plates.

To conclude, the first lens design approach, the 3D printed planar *FZP*_2_ plastic lens were built based on the FDM additive manufacturing process. In FDM printers, the lens model was created layer by layer by heating and extruding thermoplastic filament as follows. The photograph of the dielectric *FZP*_2_ model is shown in Fig. 9(f), which is obtained by ABS-M30 polymer plastic with *ε*_*r*_ = 2.76, and *tanδ* = 0.059. The intended plastic lens radiating aperture is composed of eight zones with a radius of 75 mm. The overall dimensions of the designed lens are 150 × 150 × 5 *mm*^3^. Figure 9(f) shows the proposed lens sub-zones aperture with the plastic polymer cells. There are eight sub-zones for the plastic lens antenna to achieve 30 *λ*_0_ diameter. The half-wave phase correction (with two relative permittivity (*ε*_*r*_)) phase plate has a design frequency of *f*_0_ = 60 GHz (wavelength *λ* = 5 mm). The *FZP*_2_ has a focal length $F\simeq 132\,{\rm{mm}}$ and lens aspect ratio of *F*/*D* = 0.88. These dielectric rings provide the half-wave phase correction over the entire aperture of the Fresnel-zone-plate. To solve multiple reflections issue, a lens with *h* = *λ* thickness was designed with lower permittivity dielectric rings to obtain a better transmission performance and focus. This lens configuration has the advantage of flat shape on front and back surfaces. Dipole and horn antenna are used as a lens illuminator in *yz*-plane. Two dielectric relative permittivity used in the design of the *FZP*_2_ zones typified as *ε*_*r*_ = 1.4, 2.76 and tan $\delta \simeq 0.05$. Both relative permittivities for dielectric sub-zones to satisfy the zone plate rules are designed with the optimized cube-shaped cells, with the help of (3) and (7), which are reviewed in Figs 3(b) and 4(a).

To conclude the second approach, the plastic *FZP*_4_ was fabricated using the selective laser sintering method, by on-stock PA 2200 plastic, which is a non-filled powder based on PA-12. The PA 2200 is a nylon that is selected from EOS GmbH - Electro-Optical Systems (EOS) production list. The measured permittivity value for this plastic is *ε*_*PI*_ = 3.6, which is a little bit higher than ABS-M30. The four dielectric relative permittivity used in the design of the *FZP*_4_ zones typified as *ε*_*r*_ = {1.4,2,2.7,3.6} and tan $\delta \simeq 0.06$. The dielectric ring radius of the *FZP*_4_ is extracted with (3) to achieve a quarter-wave phase correcting lens. The *FZP*_4_ planar plastic lens aperture is composed of twelve zones with the whole radius of 75 mm. The quarter-wave phase correction (with four relative permittivity (*ε*_*r*_)) *FZP*_4_ lens, is shown in Fig. 10, has a design frequency of *f*_0_ = 60 GHz, and a focal length *F* = 132 mm. As shown in Fig. 10, the realized lens is constituted by a solid ring, and three virtual permittivities created ring per full wave zone.

The simulation results show that the multi-relative permittivity zone plates hold on right permittivity tolerance which simplifies the lens design and manufacturing process. This permittivity tolerance for Polyimide (*PI*) that realized with cube-shaped cells is analyzed in Fig. 3(b). The full design process of the draft *FZP*_4_ lens is created with *PI* plastic.

## Experimental Results and Discussions

**Figure 11:**
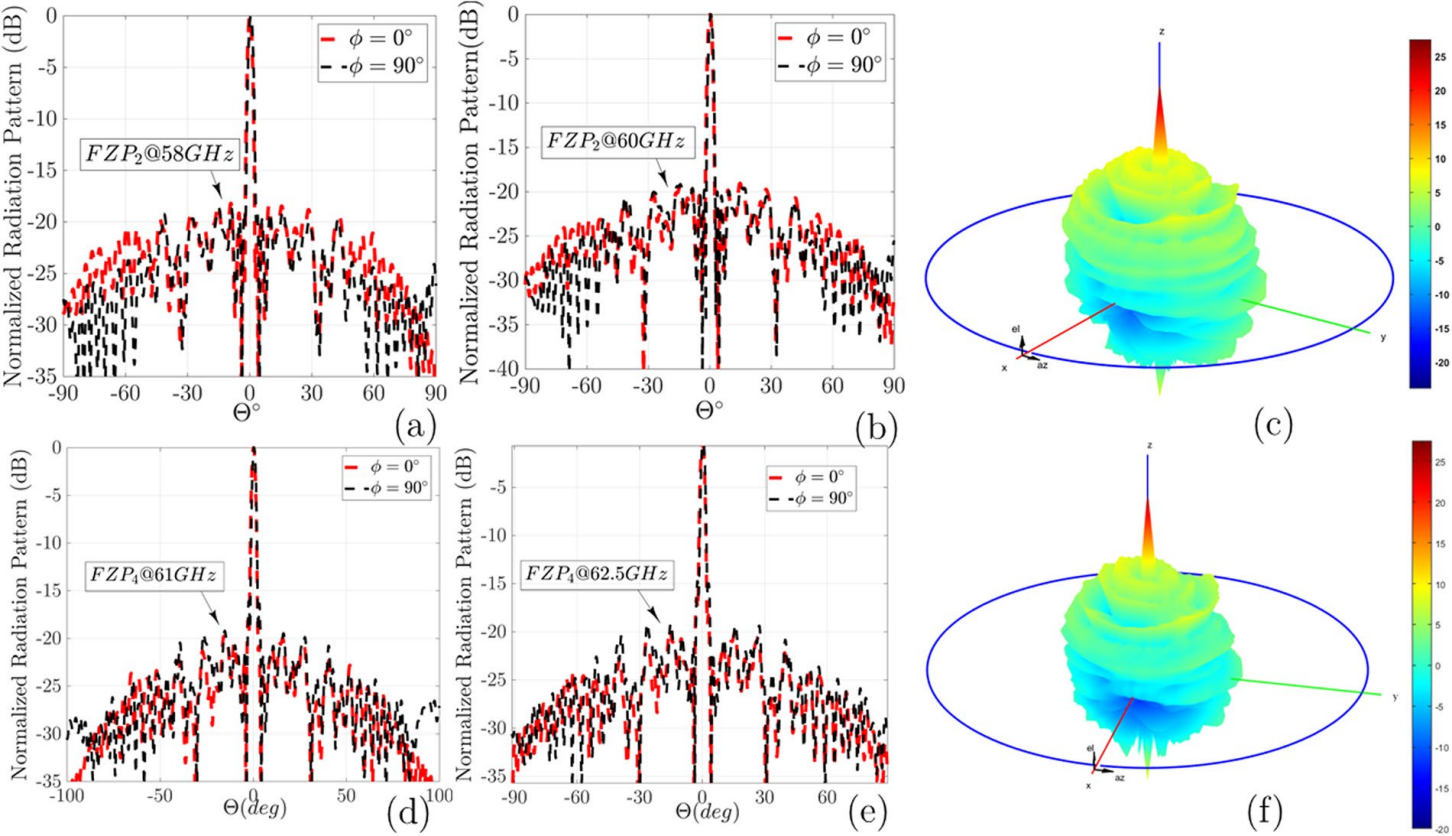
Measured radiation patterns in *ϕ* = 90°, and *ϕ* = 0° for *FZP*_2_: (**a**) 58 GHz. (**b**) 60 GHz. (**c**) 3D plots of far-field radiation patterns; Measured radiation patterns in *ϕ* = 90°, and *ϕ* = 0° for *FZP*_4_: (**d**) 61 GHz. (**e**) 62.5 GHz. (**f**) 3D.

**Figure 12:**
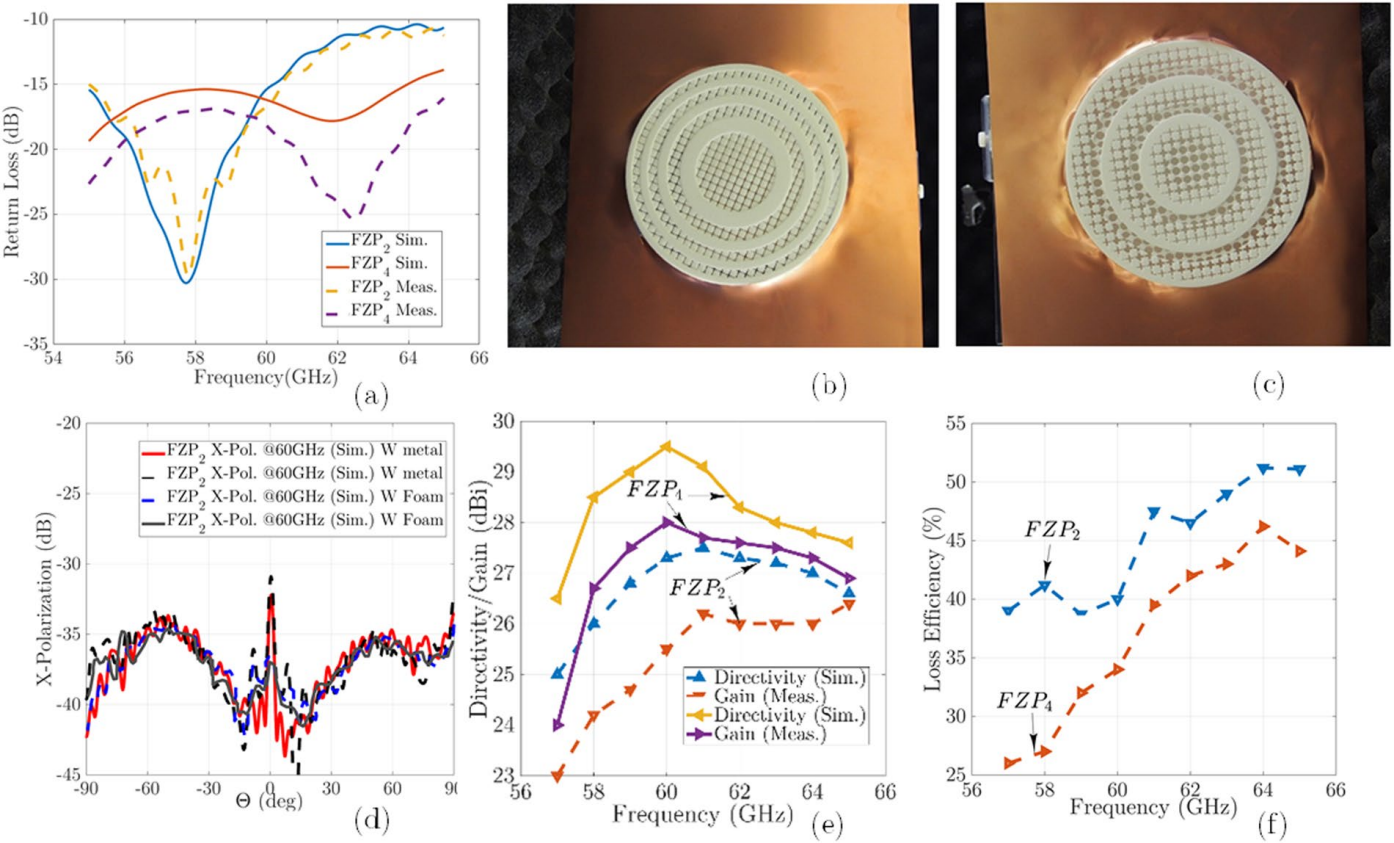
(**a**) Simulated and measured return loss results for *FZP*_2,4_ lens antennas; (**b**) Fabricated *FZP*_2_ lens with metal sheet holder; (**c**) Fabricated *FZP*_4_ lens with metal sheet holder; (**d**) Measured and simulated x-polarization level with foam and metal sheet holders; (**e**) Simulated directivity, and measured gain for *FZP*_2,4_ lens antennas with dipole illuminators (**f**) The loss efficiency for *FZP*_2,4_ lens antennas.

The measurement process for these antennas was performed in an anechoic chamber using the OML millimeter-wave standard modules with both illuminators. In this step, two customized *FZP*_2_, and *FZP*_4_ plastic lenses, Agilent E8361A PNA Network Analyzer, the OML millimeter-wave modules, a 60 GHz dipole antenna, and a V-band conical horn antenna are used as measurement components. Aligning the feeding aperture of the *FZP* lenses to the illuminator for measurements is a critical point at 60 GHz. Then, the plastic fixture is used to the fix the proposed lenses between the illuminator and reflector. The measured results for the radiation patterns for both ZPs are plotted in Fig. 11. The impedance bandwidth of the proposed FZPL antennas for dipole illuminator is measured using the Agilent E8361A network analyzer, as shown in Fig. 12, which covers the 55–65 GHz operating bandwidth. For the first *FZP*_2_ lens simulation, the maximum directivity is 27.6 dBi at 60 GHz, and the realized gain is higher than 25 dBi in 58–64 GHz. The *FZP*_2_ lens measurement results show that the maximum gain is 26.3 dBi at 60 GHz, and the measured gain is greater than 24 dBi in the 57–64 GHz impedance bandwidth. The simulated and measured 2-D and 3-D radiation patterns over the operating band for both lenses are shown in Fig. 11(a–f) for *E*(*xz*-plane)- and *H*(*yz*-plane)- planes. The dipole antenna is optimized to have *n* = 10 like radiation patterns to analyse proposed design spillover for similar diameter lens for both *n* = 10 and *n* = 61 radiation patterns. With constant diameters and analysis of the spillover and efficiency for different illuminators, the estimated *n* range for illuminators is approximated experimentally. The approximated *n* for cos^*n*^-like radiation patterns is equal to the average of *n* = 10 and *n* = 61, which is equivalent to 35. This number is very close to our analysis, as shown in Fig. 6, to achieve optimum efficiency.

For both lens prototypes, the main radiating lobe keeps pointing as a pencil beam at the boresight direction over the operating band frequency. As shown in Fig. 11, the half-power beam widths of the proposed *FZP*_2_ and *FZP*_4_ are 2.2° and 2°, and the side lobe levels are −20 dB, and −18 dB, respectively. The *FZP*_4_ measured SLLs in the *E*-plane are below −18dB at 58 GHz, and 60 GHz, respectively. The *E*- and *H*-plane radiation patterns are symmetrical because of symmetrical structure of the lenses. Figure 11 shows the normalized measured radiation patterns at 58 GHz and 60 GHz. The calculated 3D radiation patterns at 60 GHz, are shown in Fig. 11(c),(f) for *FZP*_2_ and *FZP*_4_, respectively. In the main lobe, the cross-polarization level in the *E*-plane are less than −24 dB, and −27 dB for *FZP*_2_, and *FZP*_4_, respectively. Figure 11(b),(c), and measured results show good agreement, which is support our analysis and design. Simulated and measured return loss results for *FZP*_2,4_ lens antennas for dipole illuminator are plotted in Fig. 12(a). Figure 12(b,c) shows experimental measuring setup for Fresnel lenses which are surrounded by metal sheet screen. The cross-polarization for this structure is lower than −33 dB, as shown in Fig. 12(d). The simulation analysis shows a relatively good stability of the gain over the operating frequency with a gain difference of 2 dB. The results indicate, by inserting of additional phase correction zone *q* > 2 to optimizing of the radiation pattern, much higher gain for *q* = 4 lens is achieved. According to the obtained results for both lenses, the radiation pattern is very directive, i.e., a low SLL and a narrow beamwidth, which certify the design process. The cross-polarization for the *FZP*_2_ structure is around −33 dB, which is low for all lens structures, as shown in Fig. 12(d). The proposed antennas directivity, gain, and loss efficiency calculated as shown in Fig. 12, small lobe at *θ* = 0° is observed.

During the measurement process, the intended dielectric lenses are surrounded by a metal sheet to reduce SLL for dipole feed with cos ^10^-like radiation patterns from an Omni-directional antenna. Our studies show that by using metal sheets for low n < 20 value, low SLL is obtainable. However, because of spillover for Omni illuminators by utilizing the metal sheets, the reflected waves from metal sheets around the lens will be focused at one point, and the combination of reflected and incident waves will produce small lobe in x-polarization, which is −33dB lower than the primary beam. Simulation results show that with decreasing the width of this surrounded metal sheet around *λ*_0_ at operating frequency, both low SLL and low lobe effect in x-polarization are solvable. During the simulation, the metal sheet thickness attributed to the increase in mesh number is not considered. For this reason, the simulation and measurement results with the surrounded foam sheet are also added in a revised version, which certifies our analysis as regards the combined results of reflect and incident waves. This lobe is eliminated in foam measurements, and the cross-polarization level is under −37dB, as shown in Fig. 12(d).

**Figure 13:**
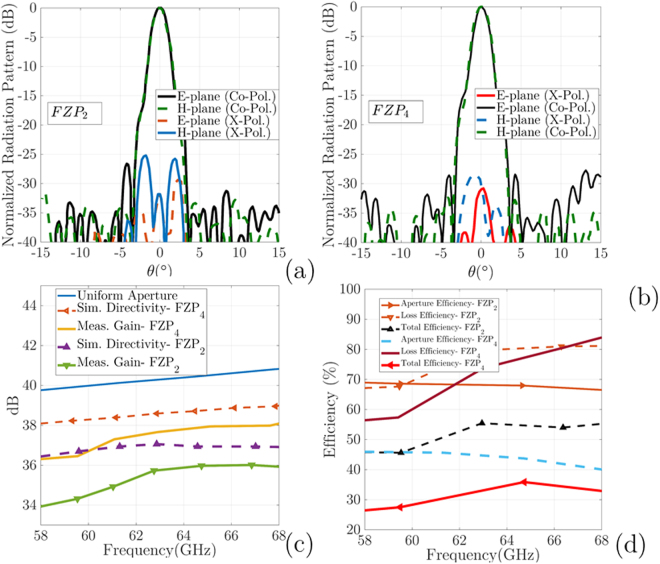
The normalized radiation patterns along E- and H- planes at 60 GHz performance with conical horn feed: (**a**) *FZP*_2_, and (**b**) *FZP*_4_ lenses; (**c**) Simulated and measured gain and directivity versus frequency; (**d**) illustration of the proposed lenses efficiency versus frequency.

Figure 13(a–d) shows a comparison between measured and simulated performances of the conical horn feed *FZP*_2_, and *FZP*_4_ lenses. Figure 13(a,b) shows the normalized radiation patterns with *cos*^*n*^-like feed conical horn at 60 GHz in E(*xz*)/H(*yz*) planes. As the obtained results, the E/H radiation patterns for conical horn feed are very directive, i.e., low SLLs and a narrow beamwidth. The cross-polarization for the *FZP*_4_ with horn feed is around −29 dB, and for the *FZP*_2_ is −25 dB. As shown in Fig. 13, the half-power beamwidth of the proposed *FZP*_2_, and *FZP*_4_ is 2°, 1.8°, respectively, and the side lobe level is −25 dB, −26 dB, respectively. The *FZP*_4_ measured SLL in the *E*-plane is below −26 dB at 60 GHz. The *E*- and *H*-plane radiation patterns are symmetrical because of symmetrical structure of the lenses, and conical horn. During the measurement process for horn feed against dipole feed, a metal sheet is replaced with a Rohacell foam (*ε*_*r*_ = 1.04) supports to consider foam base measurements also. In the main lobe, the cross-polarization level in the *E*-plane is less than −28 dB for both *FZP*_2_, and *FZP*_4_ zone plates, respectively. The proposed antenna directivity, gain, and efficiency in Fig. 13(b) and (c), and measured results show a good agreement, which validates the proposed design. As we expected, the measured gain and simulated directivity of the *FZP*_2_ lens are lower than *FZP*_4_. As shown in Fig. 13(b) the *FZP*_4_ maximum measured gain is 38 dBi at 65 GHz, whereas the *FZP*_2_ maximum measured gain is 36 dBi at 64.2 GHz with variations of less than −2dB over operating band for both of them. Figure 13(b) shows the comparison gain and directivity of both lenses with uniform aperture (orange dash-line) directivity also. In Fig. 13(c), three kinds of efficiency for conical horn feed *FZP*_2_ and *FZP*_4_ zone plates are presented as follows: aperture efficiency, loss efficiency, and total efficiency. In this study, aperture efficiency is the comparison of the maximum directivity of a uniform E-field (amplitude and phase) aperture of similar lens dimensions with the maximum simulated directivity of the lens using CST software. The measured loss efficiency is the comparison of the maximum simulated directivity with the maximum measured far-field gain of the antenna, and the total efficiency is the product of the aperture and loss. Figure 13(c) shows that the measured aperture efficiency for *FZP*_4_ is 58%. By contrast, the measured aperture efficiency of *FZP*_2_ just reaches 37%. The achieved directivity difference between the uniform aperture and FZPs indicates that a better overall efficiency for the proposed lenses with the low-SLL-optimized horn or another type of manufactural illuminator is possible. Table 1 shows the comparison of the proposed design performance to previously reported works with the homogenous medium platform.

**Table 1:** Summarized comparison of the proposed design performance compared to previous reported works.

Antenna Type	*η*_*Total*_	Technology	Lens Type	SLL(dB)	Freq. (GHz)
Metal Reflect-array^17^	30.2%	Metal Perforation	Homogeneous (Metal)	−22	75
Perforated *λ*/4 FZP^11^	33%	Plastic Perforation	Homogeneous (Plastic)	−22	30
Reflector-type *λ*/4 FZP^18^	43%	PCB	Non-Homogeneous	−20	10
Our *FZP*_4_	36%	3D-FDM	Homogeneous (Plastic)	−25	60
Our *FZP*_2_	56%	3D-SLS	Homogeneous (Plastic)	−25	60

The simulation and measurement results for these plastic zone plates certify them as an alternative prototype for conventional planar lenses. In addition, it is evident from results that additive manufacturing (ADM) process has significant potential with the combination of other technologies to contribute zone plate lens structures. It is also interesting to mention that according to our knowledge, the proposed lenses have been the first designed structures with this porosity controlling technique with cube-shaped cells, which is an alternative method to develop planar lens structures.

## Conclusion

In this paper, a new gradient refractive index FZP using dipole and horn antennas as lens illuminators have been presented. These FZP antennas in two and four phase correction sub-zones have been implemented with the homogeneous plastic material to realize inhomogeneous scheme. Two type of additive manufacturing process has been used to construct the proposed plastic lenses. The 60-GHz planar FZP lens antenna has been proposed for high-gain applications at millimeter-wave bands. The performance of the realized antennas has been measured in an anechoic chamber. Both analytical and measurement analysis show that the selection of the relative permittivity and lens fabrication with a homogenous material simplifies the realization of inhomogeneous lens schemes. The measured results have reasonably validated the proposed plastic based realization of multi-dielectric lens structures. Furthermore, it certifies the additive manufacturing (ADM) process as an alternative method to contribute zone plate lenses.
