# Author Correction: Towards Millimeter-wavelength: Transmission-Mode Fresnel-Zone Plate Lens Antennas using Plastic Material Porosity Control in Homogeneous Medium

**DOI:** 10.1038/s41598-018-28407-9

**Published:** 2018-07-03

**Authors:** Javad Pourahmadazar, Tayeb A. Denidni

**Affiliations:** National Institute of Scientific Research (INRS), Centre for Energy, Materials and Telecommunication (EMT), Quebec, Montreal H5A 1K6 Canada

Correction to: *Scientific Reports* 10.1038/s41598-018-23179-8, published online 28 March 2018

The original version of this Article contained errors.

Reference 19 was inadvertently omitted from the final version of our Article. The corrected article provides a comparison between our proposed design and that shown in reference 19 and explains how our Fresnel-Zone Plate design may overcome drawbacks in reference 19.

Additionally, there were typographical errors in Equations 8-10. The authors apologize for these errors and any confusion caused.

“In millimeter-wave band, the fabrication of illuminators that uniformly illuminates the lens surface to obtain an intended efficiency is difficult. For this reason, amplitude tapering must be considered for illumination of lens feed to obtain the desired efficiency. To accomplish this goal, two crucial factors are studied: the taper efficiency (*η*_*taper*_) and the spill-over efficiency (*η*_*SP*_). In the classic design of Fresnel zone plates, horn antennas are employed as an illuminator to feed the zone plates, the radiation pattern of the lens feeder serves a cos^n^-like function. Since the fabrication of this kind of feed at millimeter-wave band is complicated. Therefore, a new type of illuminator or cos^n^-like radiation pattern horn with low SLL must be considered for illumination. To reach this purpose, theoretical aperture efficiency versus cos^n^-like radiation pattern amplitude weighting is analyzed to obtain expected efficiency with *n* variations. The taper efficiency (*η*_*taper*_)equation for the loss of non-uniform illumination of the aperture amplitude and the spill-over efficiency (*η*_*SP*_) are given by (8) and (9), individually^15,16^:

8 $${\eta }_{taper}=\frac{1}{\pi .\,{\tan }^{2}(\frac{{\theta }_{0}}{2})}.\frac{{[{\int }_{0}^{2\pi }{\int }_{0}^{{\theta }_{0}}{|G(\theta ,\varphi )|}^{0.5}.\tan (\frac{\theta }{2})d\theta d\varphi ]}^{2}}{{\int }_{0}^{2\pi }{\int }_{0}^{{\theta }_{0}}|G(\theta ,\,\varphi )|.\,\sin (\theta )d\theta d\varphi }\,,$$

9 $${\eta }_{SP}=\frac{{\int }_{0}^{2\pi }{\int }_{0}^{{\theta }_{0}}G(\theta ,\,\varphi ).\,\sin (\theta )}{{\int }_{0}^{2\pi }{\int }_{0}^{{\theta }_{\pi }}G(\theta ,\,\varphi ).\,\sin (\theta )d\theta d\pi },$$

where G(*θ, ϕ*) is the radiation pattern, *θ* is dedicated to polar angle, and *ϕ* is the azimuthal angle^15,16^.

10 $$\begin{array}{c}G(\theta ,\,n)=\{(2n+1).\,co{s}^{n}(\theta ),\,for\,0\le \pi /2;\\ 0,\,for\,\theta  > \frac{pi}{2};\end{array}$$

Since the axially symmetric radiation pattern for illuminator is given by (10)^15^, accordingly, with increasing *n*, the proposed feed will generate high spill-over efficiency (*η*_*SP*_), and the taper efficiency (*η*_*taper*_) will decrease. Considering to the lens diameter and the lens focal length (*F*), a higher *n* for cos^n^-like radiation pattern to obtain a product of two efficiencies as total efficiency (*η*_*T*_ = *η*_*taper*_.*η*_*SP*_) is desirable. Illustration of this efficiency versus *n* value, as shown in Fig. 6, shows that the optimum point (η^*^) to obtain maximum total efficiency *η*_*T*_ = 0.81 is equal to n = 40. As shown in Fig. 6, to obtain the total efficiency between 0.31 < *η*_*T*_ < 0.71, *n* must be chosen between 10 <*n*<20 ranges. Considering to provided information for total efficiency higher than *η*_*T*_ = 0.71, *n* must be chosen between 22 and 78. For this purpose, two types of feed as illuminators to study of the designed FZPs efficiency are considered: (a) a cos^10^-like radiation pattern feed, and (b) a cos^45^-like radiation pattern feed. To achieve the first feed with cos^10^-like radiation pattern a microstrip dipole antenna is designed, and to obtain second feed with cos^40^-like radiation pattern a commercial horn antenna are considered to feed lens.”

now reads:

“Lens design criteria for the focusing devotions can be classified into two modes of illumination: transmission and reflection modes. The essential devices of each focusing setup are assigned to two central parts that can achieve them in all pieces of literature [1–19] with specific names: a lens device (focusing provider) and an illuminator/feeder/wave-launcher. Generally, all classes of lens devices for wave-focusing setup are independent of electromagnetic spectrums, and they have had their treatment scenarios and produce predetermined sub-zone permittivity techniques. However, the treatment of the proper illuminator and low-error subzone permittivity implementation is the apparent goal of all focusing mechanisms, which can be considered to guarantee the desired radiation effect in a predetermined setup with a defined focal point (F), lens diameter (D), and antenna gain parameters.

As shown in Fig. 1 (a), a Fresnel zone plate is a planar symmetric gradient index structure with a z-axis in the center. Therefore, by providing axially symmetric illumination [9] for feeding lens apertures, the zone plate’s radiation patterns should be symmetrical with high gains and high efficiency at the focusing point (P1). Therefore, we can verify that any asymmetrical zone plate’s radiation is due to permittivity estimation errors in the homogeneous design principles. To achieve this goal, an axially symmetric feed [6] or uniform feed [15, 19] based on the [15, 19] feed analysis is considered.

In millimeter-wave band over 30 GHz, the fabrication of illuminators that uniformly [15, 19] or axially symmetric form illuminates [6] the lens surface to obtain an intended efficiency is difficult [19]. For this reason, amplitude tapering must be considered for illumination of lens feed to obtain the desired efficiency for proposed lenses based on [15], and [19] feed analysis. To accomplish this goal, two crucial factors are considered based on Kildal feed analysis, and approximations in [15]: the taper efficiency (*η*_*taper*_) and the spill-over efficiency (*η*_*SP*_) [15, 19]. In the classic design of Fresnel zone plates, horn antennas are employed as an illuminator to feed the zone plates, the radiation pattern of the lens feeder serves a cos^n^-like function [6], [15], [19]. Since the fabrication of this kind of feed at millimeter-wave band is complicated [19]. Therefore, a new type of illuminator or cos^n^-like radiation pattern horn with low SLL must be considered for illumination [6], [15], [19]. To reach this purpose, theoretical aperture efficiency versus cos^n^-like radiation pattern amplitude weighting is analyzed to obtain expected efficiency with *n* variations [15, 19]. The taper efficiency (*η*_*taper*_)equation for the loss of non-uniform illumination of the aperture amplitude and the spill-over efficiency (*η*_*SP*_) are given by (Eq. 8) and (Eq. 9), individually [15], [16], [19]:

8 $${\eta }_{taper}=\frac{1}{\pi .{\tan }^{2}(\frac{{\theta }_{0}}{2})}.\,\frac{{[{\int }_{0}^{2\pi }{\int }_{0}^{{\theta }_{0}}{|G(\theta ,\varphi )|}^{0.5}.\tan (\frac{\theta }{2})d\theta d\varphi ]}^{2}}{{\int }_{0}^{2\pi }{\int }_{0}^{{\theta }_{0}}|G(\theta ,\varphi )|.\,\sin (\theta )d\theta d\varphi }\,,$$

9 $${\eta }_{SP}=\frac{{\int }_{0}^{2\pi }{\int }_{0}^{{\theta }_{0}}G(\theta ,\varphi ).\,\sin (\theta )d\theta d\varphi }{{\int }_{0}^{2\pi }{\int }_{0}^{\pi }G(\theta ,\varphi ).\,\sin (\theta )d\theta d\varphi },$$

where G(*θ, ϕ*) is the radiation pattern, *θ* is dedicated to polar angle, and *ϕ* is the azimuthal angle^15,16^.

10 $$\begin{array}{c}G(\theta ,n)=\{(2n+1).\,co{s}^{n}(\theta ),\,for\,0 < \theta  < \pi /2;\\ 0,\,\,for\,\,\theta  > \frac{\pi }{2};\end{array}$$

Since the axially symmetric radiation pattern for illuminator is given by (Eq. 10) [6], accordingly, with increasing n, the proposed feed will generate high spill-over efficiency (*η*_*SP*_), and the taper efficiency (*η*_*taper*_) will decrease [19]. Considering to the lens diameter and the lens focal length (F), a higher *n* for cos^n^-like radiation pattern to obtain a product of two efficiencies as total efficiency (*η*_*T*_ = *η*_*taper*_.*η*_*SP*_) is desirable [15, 19]. Illustration of this efficiency versus *n* value, as shown in Fig. 6, shows that the optimum point (η^*^) to obtain maximum total efficiency *η*_*T*_ = 0.81 is equal to *n* = 40 [15, 19]. As shown in Fig. 6, to obtain the total efficiency between 0.31 < *η*_*T*_ < 0.71, *n* must be chosen between 10 <n<20 ranges. Considering to provided information for total efficiency higher than *η*_*T*_ = 0.71, *n* must be chosen between 22 and 78. For this purpose, two types of feed as illuminators to study of the designed FZPs efficiency are considered: (a) a cos ^10^-like radiation pattern feed, and (b) a cos ^45^-like radiation pattern feed. To achieve the first feed with cos ^10^-like radiation pattern a microstrip dipole antenna is designed, and to obtain second feed with cos ^45^-like radiation pattern a commercial horn antenna are considered to feed lens.

Concerning structural comparison with [19], the provided lenses do not have any structural similarities or physical relationships and are just determined by the classification of their Fresnel zone plate arrangements with different applications toward two distinct bodies: planar and corrugated forms. In the case of feed types and illuminator designs, all Fresnel lenses analyses with horn antenna illuminations are similar, which is illustrated in [6] for high-frequency treatments in detail. The presented study in [6] describes the high-efficiency lens treatments while focusing on the type of illuminations such as axially symmetric feeds (Eq. 9) and lens classes. Although based on [6] studies, the results of both hard and soft material lenses are foreseeable, but we and [19] designers tried to solve manufacturing zone plate difficulties with different soft and hard plastic materials. In [19] Fresnel zone prototypes, the designers have decided to use high-efficiency luminosity in the feeding section at the expense of manufacturing problems and maintenance costs in lens platform to achieve high efficiency. However, the general purpose of our structure is concentrated to producing a cubic cell with the ability of intended permittivity control in a homogeneous environment, which is entirely dissimilar and innovative for Fresnel lens treatments.

Based on two types of radiation feed applied to both lens surfaces, as described in the next sections, lenses-out radiation has an entirely symmetrical form, a high gain, and high efficiency. Expected results compared to previously reported devices with similar type feeds, as shown in Table I, indicate the accuracy of the estimated permittivity method for phase corrector zones designed with hard plastic cube-shaped cells. Regarding the mentioned results with two distinct materials, the proposed design scheme has already answered the earlier problems with low efficiencies in hard plastic slabs (see Table I). Also, it has responded to the issues of manufacturing and keeping in services for the similar Fresnel-type lenses in [19] with soft foam materials.”

The original Figure 6 legend,

“Illustration of η_taper_, η_SP_ and η_total_ efficiency over the amplitude weighting generated by cos^n^-like illumination.”

now reads:-

“Graphical illustration of η_taper_ (Eq. 8), η_SP_ (Eq. 9), and η_total_ efficiency [19] over the amplitude weighting generated by cos^n^-like illumination [6, 19].”

